# Biliary involvement in liver metastases: long-term experience with biliary biopsy from a single center

**DOI:** 10.1590/0100-3984.2020.0004

**Published:** 2021

**Authors:** Riccardo Inchingolo, Massimiliano Nestola, Thiago Franchi Nunes, Stavros Spiliopoulos, Michele Nardella

**Affiliations:** 1 Division of Interventional Radiology, Department of Radiology, Madonna delle Grazie Hospital, Matera, Italy.; 2 Interventional Radiology Unit, “F. Miulli” Regional General Hospital, Acquaviva delle Fonti (BA), Italy.; 3 Division of Interventional Radiology, Department of Radiology, Hospital Universitário Maria Aparecida Pedrossian da Universidade Federal de Mato Grosso do Sul (HUMAP-UFMS), Campo Grande, MS, Brazil.; 4 2nd Radiology Department, School of Medicine, National and Kapodistrian University of Athens, Athens, Greece.

**Keywords:** Liver, Biliary tract diseases, Radiology, interventional/methods, Biopsy/methods, Fluoroscopy/methods, Fígado, Doenças biliares, Radiologia intervencionista/métodos, Biópsia/métodos, Fluoroscopia/métodos

## Abstract

**Objective:**

To investigate long-term results of biliary biopsy performed with transluminal forceps in the setting of metastatic biliary involvement.

**Materials and Methods:**

Between September 2014 and June 2019, 25 patients-18 males (72%)-with a mean age of 65 ± 15 years, underwent 26 biliary biopsy procedures with a dedicated forceps system. All patients presented with obstructive jaundice that was suspected of being malignant and underwent pre-procedural magnetic resonance cholangiopancreatography. The biopsies were performed during percutaneous placement of an internal-external biliary drainage catheter, under fluoroscopic guidance.

**Results:**

The technical success rate was 96% (corresponding to 25 of the 26 procedures). The histological diagnosis was inflammatory biliary stricture in five cases, pancreatic adenocarcinoma in six, liver metastases from colorectal cancer in eight, and hepatocellular carcinoma in three, the biliary mucosa being categorized as normal in three cases. In one case, the sample was considered insufficient and the procedure was successfully repeated, after which a diagnosis of pancreatic adenocarcinoma was made. Over a follow-up period of 6-48 months, there were five false-negative results: two findings of inflammatory biliary stricture were later identified as liver metastases from breast and gastric cancer, respectively; and all three patients in which the biliary mucosa was categorized as normal were subsequently diagnosed with metastatic hilar lymph nodes. The procedure was found to have a sensitivity of 77%, a specificity of 100%, and an overall accuracy of 80%. The complication rate was 11.5% (mild, transient hemobilia occurring in three cases).

**Conclusion:**

Percutaneous transluminal forceps biopsy is a safe, effective, minimally invasive procedure for histological characterization in patients presenting with obstructive jaundice due to a non-primary biliary tumor.

## Abstract (pt)

**Objetivo:**

Investigar os resultados a longo prazo da biópsia endobiliar realizada com um pinça tipo fórceps transluminal no diagnóstico de neoplasia biliar metastática.

**Materiais e Métodos:**

Entre setembro de 2014 e junho de 2019, 25 pacientes - 18 homens (72%), com idade média de 65 ± 15 anos) - foram submetidos a 26 procedimentos de biópsia endobiliar com um conjunto dedicado. Todos os pacientes apresentaram icterícia obstrutiva, suspeita de malignidade e colangiorressonância pré-procedimento. Os procedimentos foram realizados durante o posicionamento percutâneo da drenagem biliar interna-externa, sob orientação fluoroscópica.

**Resultados:**

A taxa de sucesso técnico foi de 96% (25 casos), com diagnóstico histológico de estenose benigna (inflamatória) em cinco casos, adenocarcinoma pancreático em seis casos, metástases hepáticas retais no cólon em oito casos, carcinoma hepatocelular em três casos e de mucosa biliar normal em três casos. Em um caso a amostra foi considerada insuficiente pelo patologista (um adenocarcinoma pancreático) e o procedimento foi repetido com sucesso. O seguimento de 6 a 48 meses mostrou cinco casos falso-negativos, em particular dois casos de metástases hepáticas retais sem cólon (câncer de mama e gástrico) e três linfonodos hilares metastáticos. A análise estatística revelou sensibilidade de 77%, especificidade de 100% e precisão geral de 80%. A taxa de complicações foi de 11,5% (três casos com hemobilia transitória).

**Conclusão:**

A biópsia biliar transluminal realizada com pinça tipo fórceps é um procedimento minimamente invasivo, seguro e eficaz para caracterização histológica em pacientes que apresentam icterícia obstrutiva no diagnóstico de neoplasia biliar metastática.

## INTRODUCTION

Despite the existence of noninvasive modern imaging systems, such as ultrasound, computed tomography (CT), and magnetic resonance cholangiopancreatography (MRCP), biliary strictures often pose a diagnostic challenge and therapeutic dilemma because of their small dimensions and nonspecific imaging findings, which can make it difficult to distinguish between benign and malignant obstruction^(1-5)^. Morphologically, biliary strictures can be classified as intrinsic or extrinsic. More specifically, most such strictures are associated with hilar cholangiocarcinoma, although in 10-25% of cases they are associated with other malignancies or benign lesions (intraductal metastasis, hepatocellular carcinoma, pancreatic adenocarcinoma, duodenal cancer, or liver metastasis), which may cause jaundice for extrinsic compression or intraductal growth, thus mimicking the clinical and radiological pattern of hilar cholangiocarcinoma^(6)^. The management of resectable cholangiocarcinoma relies on bile duct excision, whereas other malignant lesions, such as metastatic lymph nodes or liver metastases, and benign strictures should not be treated surgically.

The current European Society for Medical Oncology (ESMO) guidelines specify that histological confirmation and immunohistochemistry are mandatory prior to deciding on a treatment strategy, in order to choose the appropriate treatment, and recommend endoscopic retrograde cholangiopancreatography (ERCP)-guided core biopsies or brush cytology and endoscopic ultrasound-guided fine needle aspiration as the tissue acquisition techniques^(7,8)^.

Percutaneous transhepatic biliary drainage (PTBD) is currently the treatment of choice in patients with biliary stent occlusion after the failure of ERCP or those with hilar strictures^(9)^. Percutaneous transluminal forceps biopsy (PTFB) of the bile duct performed during PTBD was first reported in 1980^(10)^, and several studies have since demonstrated its safety and sensitivity, especially in cases of cholangiocarcinoma. Nevertheless, this technique is neither cited nor recommended in the ESMO guidelines^(8)^, probably because all published reports have involved only a small number of cases and the level of evidence therefore remains low^(1,2,10-14)^. To our knowledge, there have been no studies analyzing the safety and efficacy of PTFB in patients with metastatic biliary involvement.

## MATERIALS AND METHODS

**Figure 1:**
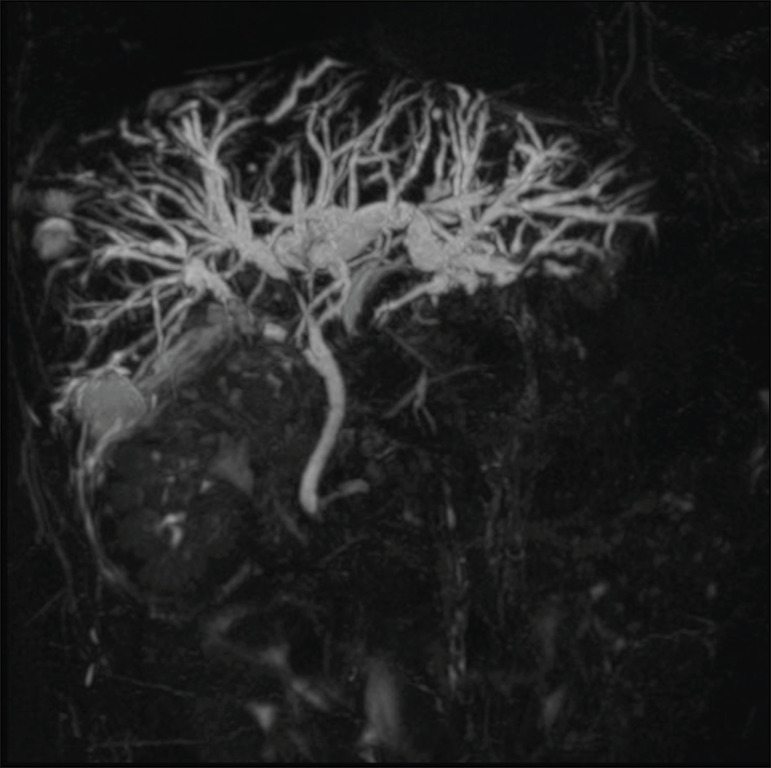
Hilar metastatic biliary stenosis. A 60-year-old man, who had previously undergone left hemicolectomy for adenocarcinoma and multiple liver resection for metastases, presented with obstructive jaundice. MRCP showed the presence of hilar stenosis with intrahepatic bile duct dilatation.

This was a retrospective, single-center, single-arm study investigating the safety and efficacy of PTFB of non-primary biliary lesions during PTBD, using a dedicated transluminal biliary access and biopsy forceps set (BBFS; Cook Medical, Bloomington, IN, USA). We reviewed imaging and medical records of 65 patients who underwent PTFB during PTBD between September 2014 and June 2019. The 65 patients underwent a total of 66 biopsies, and 40 patients were found to have cholangiocarcinoma. The remaining 25 patients-18 males (72%)-with a mean age of 65 ± 15 years, were included in our study population. The 25 patients, all of whom suffered from obstructive jaundice, underwent a total of 26 biopsies during PTBD, and the specific biopsy forceps set was used in all of the procedures. All of the patients had previously undergone MRCP to determine the degree of stenosis and to depict the biliary anatomy more accurately (Figure 1).

The primary endpoint of the study was technical success, defined as the successful acquisition of at least one tissue sample deemed sufficient by the pathologist, and the rates of major and minor procedure-related complications. The secondary endpoints were the sensitivity, specificity, and overall accuracy of the procedure for the characterization of malignancy. Complications were classified as major or minor according to the Society of Interventional Radiology (SIR) recommendations for percutaneous transhepatic biliary procedures^(15)^.

Statistical analysis was performed with the SPSS statistical software package, version 21.0 (IBM Corp., Armonk, NY, USA). Values of *p* < 0.05 were considered statistically significant.

### Procedure

All procedures were performed in the angiography suite. Local anesthesia (lidocaine 2%) was administered at the puncture site. If necessary, the procedure was performed under conscious sedation with intravenous fentanyl, midazolam, or a combination of the two. The PTBD was performed in accordance with the SIR recommendations^(15)^.

**Figure 2:**
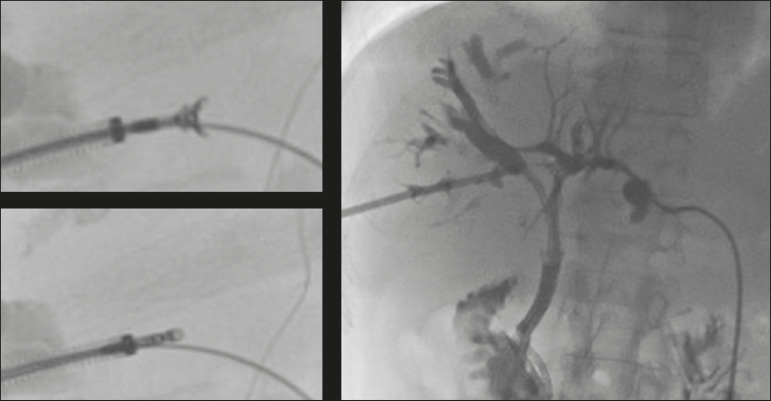
Hilar metastatic biliary stenosis (same patient depicted in Figure 1). During PTBD, the patient underwent intraluminal biliary biopsy. The forceps were first opened, and then closed, within the tissue. The procedure ended with the bilateral placement of internal-external drains. The pathologist confirmed peribiliary diffusion of metastatic adenocarcinoma, consistent with colorectal cancer.

**Figure 3:**
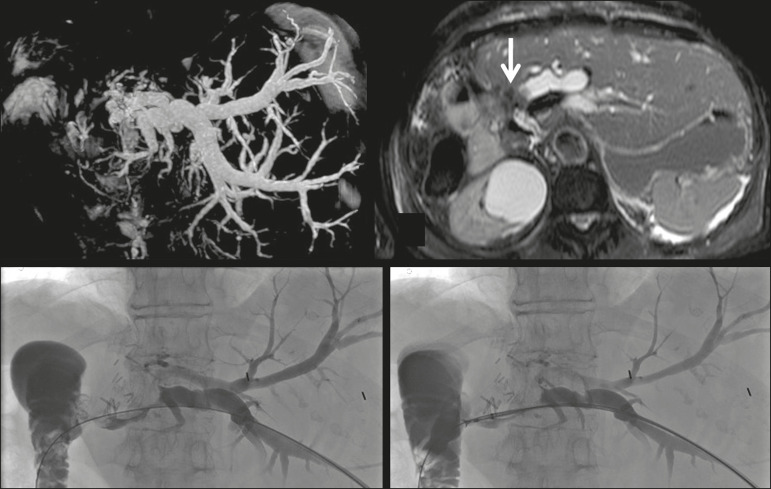
A 76-year-old woman, who had undergone right hepatectomy for metastases from colorectal cancer 2 years prior, presented with obstructive jaundice. MRCP showed stenosis of the left hepatic duct with intrahepatic bile duct dilatation, together with some amount of tissue within the duct (arrow). Biopsy samples were collected from the proximal and distal portions of the duct. The pathologist confirmed the presence of biliary metastases from adenocarcinoma of the large bowel.

Intraluminal biopsy was performed using the cross and push technique as previously described^(16)^. In brief, after percutaneous transhepatic access had been obtained with the 22G needle and 6F sheath in the Neff percutaneous access set (Cook Medical), the biliary obstruction was crossed using a 5F biliary manipulation curve catheter (Torcon NB Advantage; Cook Medical) and a 0.035-in. hydrophilic guidewire (Roadrunner; Cook Medical). Subsequently, a 7F × 30 cm sheath (Flexor; Cook Medical) was positioned within the stenosis, over a 0.035-in. support guidewire (Amplatz Super Stiff; Cook Medical), the tip of which was positioned within the duodenum. The support guidewire was left in place, for safety, and the forceps were then inserted along the guidewire, through the sheath, after which they were advanced into and opened within the lesion under fluoroscopic guidance, the sheath being used for support^(15)^, as depicted in Figures 2 and 3.

Up to five samples were taken from each lesion. The samples were fixed with formalin and sent to the pathology department for analysis. Following biopsy, an 8.5F internal-external biliary drain was positioned over the support wire.

## RESULTS

The median number of biopsy samples obtained was 3 (range, 3-5). Among the 25 patients evaluated, the obstruction was in the hilar/proximal portion of the bile duct in 17 (68%), in the middle portion in 2 (8%), and in the distal portion in 6 (24%). Among the 26 procedures evaluated, the quantity of tissue sampled was sufficient to allow the histological diagnosis in 25, which translates to a technical success rate of 96%. In the one case in which the pathologist considered the sample insufficient to allow histological characterization, the biopsy was successfully repeated and a diagnosis of pancreatic cancer was made. The histological diagnosis included liver metastases from colorectal cancer in eight patients (32%), pancreatic adenocarcinoma in six (24%), hepatocellular carcinoma in three (12%), and inflammatory biliary stricture in five (20%), the biliary mucosa being categorized as normal in the three remaining patients (12%). There were five false-negative results. The three patients in which the biliary mucosa was categorized as normal were later found to have metastatic lymph nodes causing hilar compression. The other two false-negative results were findings of inflammatory biliary stricture in patients who were subsequently diagnosed with liver metastases from breast and gastric cancer, respectively.

Percutaneous intraluminal biopsy using the specific access and biopsy forceps set had a sensitivity of 77%, a specificity of 100%, and an overall accuracy of 80%. In four cases, it posited a diagnosis of pancreatic cancer following inadequate tissue sampling from ERCP-guided trans-papillary forceps biopsies.

There were no major complications related with any of the procedures evaluated. However, three patients (11.5%) developed hemobilia, manifesting as right quadrant pain and blood draining from the catheter, without a significant drop in hemoglobin (< 2 g/dL). In all cases, transfusion or prolonged hospitalization was not required, because the hemobilia was self-limited, resolving within 48 h.

## DISCUSSION

The diagnostic imaging methods currently available have been shown to have high sensitivity and specificity for the detection and localization of biliary strictures^(1-3)^. Nevertheless, it is not always feasible to characterize such strictures.

Cholangiocarcinoma is the most common cause of malignant biliary obstruction, typically showing an intrinsic growth pattern. However, in 10-25%^(6)^ of cases, benign lesions or other malignancies (pancreatic and duodenal cancer and liver metastasis) can cause jaundice resulting from extrinsic compression of the biliary tree or, in a smaller proportion of cases, from intraductal involvement. In addition, in this era of “tailored” drug therapies, the ESMO guidelines state that tissue specimen collection is the best approach in terms of specificity, further stating that histological characterization and immunohistochemistry are mandatory prior to any treatment planning and determination of prognosis^(7-9)^.

Percutaneous fine needle-aspiration biopsy, under ultrasound or CT guidance, has been shown to be less accurate in the biliary system than at other locations, because of the small size of biliary lesions and poor visualization of such lesions as biopsy targets^(5)^. Endoscopic or percutaneous intraluminal techniques are generally used in order to collect tissue specimens through bile aspiration, brushing cytology, forceps biopsy, or ultrasound-guided fine needle aspiration^(1)^. Although bile aspiration and brushing cytology, performed during PTBD or ERCP, have been proven to be safe and effective, there have been reports showing that they have low (35-61%) sensitivity^(16)^.

After failed ERCP or in cases of hilar strictures, PTBD is the only treatment available for obstructive jaundice, providing technical and clinical results comparable or superior to those achieved with endoscopy, as well as allowing access to the intrahepatic and extrahepatic bile ducts, in order to introduce various biopsy instruments. The use of PTFB was first reported in 1980^(10)^, and it has since been used successfully in many cases, with a reported sensitivity that ranges from 78% to 93% and can even be as high as 96% in selected cases of cholangiocarcinoma^(1,12)^. Nevertheless, the ESMO guidelines do not include a recommendation regarding the use of PTFB^(8)^, probably due to the low level of evidence in literature. In addition, previous studies have demonstrated that PTFB has low sensitivity and specificity in cases of biliary strictures caused by extrinsic lesions^(1,4,12,17)^.

In the present retrospective analysis, PTFB using the specific access and biopsy forceps set had a sensitivity of 77%, a specificity of 100%, and an overall accuracy of 80%. These data are in agreement with those of other recently published studies, in which the reported sensitivity of the procedure ranged from 75% to 92%^(1,15,17)^. In a large retrospective analysis of 271 percutaneous biliary biopsies of primary biliary malignancies and secondary liver tumors, Park et al.^(5)^ reported that PTFB had a sensitivity of only 77.2% and an overall accuracy of only 78.9%, those lower rates being attributable to the fact that false-negative results were obtained in 57 (21%) of the 271 procedures evaluated in that study, compared with 5 (20%) of the 25 cases procedures evaluated in the present study.

The false-negative results in our sample were subsequently proven to be extrinsic compression by metastatic hilar lymph nodes in three cases and liver metastases from breast and gastric cancer, respectively, in the other two cases. These data are in agreement with those reported in literature. In particular, Sato et al.^(18)^ demonstrated that the sensitivity and efficacy of PTFB are lower when biliary malignant strictures are caused by extrinsic lesions than when they are caused by intrinsic or infiltrative lesions, probably because those authors obtained biopsy specimens only from the mucosa and superficial part of the fibromuscular layer of the duct. They therefore suggested that forceps biopsy is less helpful in characterizing extrinsic tumors or tumors involving the external wall of the bile duct.

To our knowledge, Terasaki et al.^(19)^ are the only authors who reported a sensitivity of 100% for forceps biopsy, although their study sample comprised only six patients-five with metastatic disease and one with cholangiocarcinoma. Park et al.^(5)^ suggested that the results of biopsy in metastatic disease are dependent on the depth to which an extrinsic malignancy has infiltrated the bile duct wall, indicating the variable degree of infiltration of the biliary wall layers as the most important predictor of a false-negative result. In addition, Estrella et al.^(7)^, in a prospective analysis, demonstrated that intrabiliary growth is significantly more common in patients with liver metastases from colorectal cancer than in those with other types of metastatic tumors (10.6% vs. 1.9%). This is in accordance with our results, given that, in our sample, PTFB correctly diagnosed all cases of liver metastases from colorectal cancer, whereas two of our false-negative results were in cases later diagnosed as liver metastases from breast and gastric cancer, respectively. In view of these considerations, the high rates of sensitivity and accuracy of PTFB become more important because of the major advances made in the development of individualized drug therapy in the setting of palliative and adjuvant treatment of colorectal cancer.

No major complications were observed in our sample, although a minor complication (mild hemobilia that was self-limited, resolving within 48 h) was seen in three cases (11.5%). In the literature, there is only one report of a major complication after PTFB, Park et al.^(5)^ reporting the case of a patient who developed hemobilia that required transarterial embolization. The reported rates of minor complications after PTFB range from 4.0%^(12)^ to 37.5%^(1)^. The most common minor complications are biloma and hemobilia^(1,4)^.

The complication rate associated with PTBD varies depending on the pre-procedural patient status, the diagnosis, and the degree of bile duct dilatation. According to Burke et al.^(20)^, hemorrhage/hemobilia after PTBD have been reported in 2.5% of cases and the Cardiovascular and Interventional Radiological Society of Europe standards of practice state that the overall procedure threshold for all major complications of PTBD should be 10%. The wide range of rates of minor complications reported in literature could be related to the neoplastic biliary involvement itself and to the degree of vascularization of the lesions. Therefore, the question of whether the hemobilia reported is related to the biopsy or to the PTBD itself remains unanswered.

Our study has some limitations. Because it was a retrospective study, it was subject to the biases inherent to studies of that nature. In addition, the lack of a control group precluded direct comparison with endoscopic modalities. Another limitation is the small number of patients evaluated. A study involving a larger patient sample would have provided a more robust statistical analysis for the identification of factors associated with misdiagnosis.

In conclusion, PTFB with the forceps biopsy set employed here is a safe and accurate procedure, with high sensitivity, specificity, and accuracy, as well as a low complication rate. Therefore, PTFB during PTBD placement should be considered a valid diagnostic tool when histological characterization is required.
